# Targeted axillary dissection using Magseed magnetic bead in female patients with breast cancer

**DOI:** 10.1016/j.clinsp.2026.101105

**Published:** 2026-09-07

**Authors:** Daniel Toman, Ilker Sengul, Anton Pelikán, Demet Sengul, Otakar Kubala, Jiří Prokop, Markéta Pernicová, Lubomír Tulinský, Peter Ihnát, José Maria Soares-Junior

**Affiliations:** a Department of Surgery, University of Ostrava, Faculty of Medicine, Ostrava, Czech Republic; b Department of Surgery, University Hospital in Ostrava, Ostrava, Czech Republic; c Thyroidology Unit, Faculty of Medicine, Giresun University, Giresun, Turkey; d Division of Endocrine Surgery, Faculty of Medicine, Giresun University, Giresun, Turkey; e Department of General Surgery, Faculty of Medicine, Giresun University, Giresun, Turkey; f Faculty of Humanities, Tomas Bata University in Zlin, Zlin, Czech Republic; g Department of Pathology, Faculty of Medicine, Giresun University, Giresun, Turkey; h Department of Imaging Methods, University Hospital Ostrava, Ostrava, Czech Republic; i Laboratório de Ginecologia Estrutural e Molecular (LIM-58), Disciplina de Ginecologia, Departamento de Obstetrícia e Ginecologia, Hospital das Clínicas, Faculdade de Medicina, Universidade de São Paulo, São Paulo, SP, Brazil

**Keywords:** Breast neoplasms, Neoadjuvant therapy, Axilla, Dissection, Pathology

## Abstract

**Introduction:**

The optimal surgical management of Lymph Nodes (LNs) following Neoadjuvant Systemic Therapy (NACT) remains a subject of clinical debate. Targeted Axillary Dissection (TAD) aims to improve staging accuracy by identifying the initially metastatic node. The prerequisite for TAD is the identification of the initially most prominent pathological nodule in the axilla before the start of treatment.

**Methods:**

This study presents a retrospective observational study of breast cancer after Neoadjuvant Systemic Therapy (NACT), from 2021 to 2024. The study primarily focused on the diagnosis of LN conditions and the surgical procedures performed. This study included n = 58 female patients with biopsy-proven cN1 breast cancer.

**Results:**

The patients with initially positive axillary LNs were evaluated. Before the start of NACT, the biopsy of the pathological node was performed and also labeled with a Magseed magnetic marker. After NACT, a standard sentinel lymph node protocol was performed with preoperative lymphoscintigraphy with Technetium colloid. Significantly, there was never a case in which a labeled node was negative while metastases were in other nodes. In 4/58 (6.9%) cases, the common sentinel nodes were negative, while the labeled TAD node was positive.

**Conclusion:**

The labeled node appears to represent the status of LNs more accurately after NACT than conventional ones. The metastatic nodes can be spared with axillary dissection using neoadjuvant treatment and TAD; however, the long-term oncologic outcomes of this approach are not yet reliably known.

## Highlights

• Magseed TAD improves staging accuracy over SLNB in post-NACT breast cancer.
• Magseed ensures detection for precise target lymph node removal.
• TAD allows safe avoidance of ALND in ycN0 patients, reducing morbidity.

## Introduction

Breast Cancer (BC) is the most common malignant neoplasm in women. As the incidence increases worldwide, so does the prevalence. In the Czech Republic, the prevalence reached women in 2018, representing an increase compared to 2008 (58,296 women). Every year, more than women are diagnosed with breast cancer in the Czech Republic due to the mammography screening program. Notably, most newly diagnosed cases are in stages I–II, which consequently means a more favorable prognosis and a decreasing mortality rate.^1^

Neoadjuvant Systemic Therapy (NACT) is utilized to enable more adequate surgical procedures targeted to specific patients with the expectation of improving the prognosis of oncological disease. NACT is typically indicated for patients who can be expected to respond positively to chemotherapy. These often include patients whose immunohistochemical tumor profile has low or negative Estrogen Receptor (ER) and Progesterone Receptor (PR) status, high-grade tumors, high Ki-67 expression, and HER2+ status. An essential step before starting any treatment, whether oncological or surgical, is to mark the primary location of the tumor with a marker if Breast-Conserving Surgery (BCS) is expected. Similarly, in the case of a positive axillary lymph node LN (cN1), it should be marked with a marker in order to avoid more extensive axillary dissection in patients who are cN0 after NACT therapy. According to NCCN guidelines for the treatment of Breast Cancer (BC), in patients who have been found to have metastatic positive axillary Lymph Nodes (LNs) prior to preoperative systemic therapy, Sentinel LN (SLN) biopsy has false negative results when performed after preoperative systemic therapy, which can be improved by marking and subsequently removing the initially biopsied node (Targeted Axillary Dissection, TAD) or by using dual indicators or obtaining sentinel nodes.^2^^,^^3^

Targeted Axillary Dissection (TAD) was introduced to improve axillary staging in patients with breast cancer after NACT therapy. Frankly, TAD consists of an SLN Biopsy (SLNB) and removal of the initially metastatic axillary lymph node. In addition, the metastatic LN is verified by Core-Cut Biopsy (CCB) and marked prior to NACT therapy with a marker to facilitate subsequent surgical extirpation, due to the possibility of LN regression after NACT therapy (Table 2). As such, false negativity of SLN means that metastases are found in non-sentinel nodes even though no tumor cells were detected in SLN. However, with fewer than three sentinel nodes, SLNB carries a risk of FN exceeding. Therefore, TAD helps us reduce the False Negativity Rate (FNR) of SLN by safely removing all pathological nodes from the axilla in cases of negative histological findings in SLN and positive findings of metastatic cells in the marked node during TAD.^4^

Notably, the use of MS (Magseed®, Endomagnetics Ltd, Cambridge, United Kingdom).^5^ in the localization of non-palpable breast lesions with probe detection has been proven as equally beneficial as the stereotactic localization technique using a clip. Herein, MS is a stainless-steel bead measuring mm and is inserted into the LN using an 18-gauge needle, usually under ultrasound guidance. Of note, detection is performed using a SentiMag® magnetometric probe (Endomagnetics Ltd, Cambridge, United Kingdom), which generates an alternating magnetic field to magnetize the iron-containing seed temporarily. Crucially, the stability of MS after marking in soft tissues has gradually increased. Since 2020, MS has been approved for insertion into soft tissues without time restrictions, which is used to mark the Target LN (TLN) before NACT. Herewith, we present a retrospective cohort of patients who underwent TAD at our facility between 2021 and 2024.^6^^,^^7^

## Methods

### Study design and setting

This is a single-center, retrospective study evaluating patients who underwent surgery between 2021 and 2024 at the Surgical Clinic of the University Hospital in Ostrava, Ostrava, Czech Republic. This prospective clinical cohort study received approval from the hospital's Ethics Committee at the University Hospital in Ostrava (Protocol n° 371/2021). This study was conducted in accordance with the principles of the Declaration of Helsinki and adheres to the Strengthening the Reporting of Observational Studies in Epidemiology (STROBE) guidelines, and all patients provided written informed consent before enrollment.

### Data collection and patient management

Data was collected from the patient's first examination and from clinical, radiological, and pathological reports. The subsequent treatment procedure was discussed individually for each patient by a Multidisciplinary Team (MDT). The breast cancer had been diagnosed by mammography or sonography and confirmed by biopsy and pathology. Additionally, the axillary LN metastasis was diagnosed by sonography and confirmed by core-cut biopsy and pathology. Subsequently, according to MDT recommendations, NACT therapy was indicated. Afterward, the index LN(TLN) was marked with a Magseed magnetic seed, and where required, the primary tumor was also marked when indicated for Breast-Conserving Surgery (BCS). NACT therapy lasted for months. After NACT, the clinical response of the cancer and axilla was evaluated both by physical examination and sonography. Another consultation was held by the Multidisciplinary Team (MDT) to re-stage and assess the findings in both the breast tumor and lymph nodes. At this critical juncture, patients were indicated for breast tumor surgery, either Breast-Conserving Surgery (BCS) or Mastectomy (ME), and axillary surgery in the form of SLNB + TAD.

### Surgical procedure and histopathology

A colloid marked with the radioactive isotope Technetium-99 m was used for SLNB, following a one-day protocol. Afterward, the operation began with a standard breast procedure, either BCS or ME, followed by SLNB detection with a gamma probe and TAD using a SentiMag magnetic probe. After extirpation, the detected nodes were sent for histopathological examination. It is essential to note that no frozen section examination had been performed. Based on the characteristics of the tumor and residual disease, adjuvant breast radiotherapy, regional LNirradiation, chemotherapy, anti-HER2 therapy, or endocrine therapy was subsequently applied postoperatively according to MDT recommendations.

## Results

Between 2021 and 2024, a total of patients underwent TAD surgery at our facility. The mean age was 51.8-years (range 30–74 years). The mastectomy procedure was performed in 34 patients (58.6%), while breast-conserving surgery was performed in 24 (41.4%). The immunohistochemistry characteristics of the tumors are shown in Table 1. Herein, no complications were reported during the ultrasound-guided introduction of Magseed beads into the lymph nodes. All 58 cases underwent neoadjuvant oncological treatment. The fifteen patients (25.9%) exhibited complete regression, and 43 (74.1%) showed a partial clinical response in the breast, as determined by ultrasonographic examination. In 12 cases (20.7%), clinical or sonographic complete regression of the axillary lymph nodes was observed, whereas in 46 cases (79.3%), only partial regression was observed. In all 58 cases (100.0%), Magseed was detected in the LN by sonography. Dual marking, for example, with blue dye, had not been used. The LN marked with Magseed was always found intraoperatively in all 58 patients (100.0%) using the SentiMag probe. SLN was detectable in 54 of 58 cases (93.1%). In 2 of 58 cases (3.4%), there was diffuse metastatic involvement of the axilla, where the radiopharmaceutical probably did not penetrate the encapsulated node. In 2 of 58 patients (3.4%), complete regression was observed, with no metastases found even in the marked LN. The FNR, metastases found in a non-sentinel labeled node even though no tumor cells were detected in the sentinel node, was observed in 4 of 58 (6.9%). A sum of 42 of 58 (72.4%) had histologically evident signs of regression in the LN, classified as Chevalier I‒III. Of the 58 patients, 16 (27.6%) exhibited only minimal histological regression changes in the LN, as classified by Chevalier IV. As such, pCR Chevalier I in LN was observed in 24 of 58 (41.3%). Complete Axillary LN Dissection (ALND) was not performed in any case. All the patients after breast-conserving surgery underwent further breast radiotherapy according to the postoperative MDT seminar, and also consolidation radiotherapy of the axillary area. The individual nature of the tumor's immunoprofile and the patient's wishes, who is properly informed about the possible risks of radiotherapy and refusal of treatment, are also decisive.

**Table 1:** The immunohistochemistry characteristics of the tumors.

The immunohistochemistry characteristics	n (%)
Luminal A	22 (38.0%)
Luminal B Her-2 positive	10 (17.2%)
HER-2 nonluminal	7 (12.0%)
Tripple negative	19 (32.8%)

**Table 2:** The rate of regression after NACT according to Chevalier.

Grade	n (%)
Grade I Complete regression	24 (41.3%)
Grade II Persistence of in situ component, invasive component missing	8 (13.8%)
Grade III Residual invasive tumor, clear regressive changes after therapy	10 (17.2%)
Grade IV Only minimal regressive changes, invasive tumor persists	16 (27.7%)

NACT, Neoadjuvant Systemic Therapy.

## Discussion

The status of lymph nodes is a strong prognostic factor and often a decisive criterion for the indication of surgery and adjuvant radiotherapy, and therefore, it is desirable to accurately determine the situation as much as possible diagnostically. It is unacceptable to consider all ultrasonographically suspicious lymph nodes as metastatic (cN1 according to the TNM classification); therefore, unclear findings must be verified by Core Needle Biopsy (CCB). Patients without histological evidence of metastasis should be classified as cN0 according to the TNM classification. When performing a Sentinel LN Biopsy (SLNB) alone, only one or two SLN are typically found, even when using a combined detection method. However, when there are fewer than three SLN. The clinical utility of removing additional nodes “blindly” to obtain at least three “sentinel” nodes is disputable, as these would no longer be sentinel nodes in the true sense of the word but rather randomly found nodes whose diagnostic value is unclear. FNR of the SLN leads to underestimation of the stage and potentially the abandonment of oncological treatment, where such treatment is entirely appropriate. Of note, there are several mechanisms for the occurrence of false negativity.

A greater distance between the active node and the incision site may lead to low activity detected by the scintillation probe. As such, if an active node is subsequently found in a more accessible location, it may be misidentified as a sentinel node. Therefore, the fifth mechanism may involve the regression of an already established nodular primary metastasis in the sentinel node, with persistence of metastasis in other nodes in the chain. If only the first node with disappeared metastasis, which may have relatively high activity in the surgical field, is removed, the phenomenon of false-negative sentinel node will occur again. Critically, TAD significantly reduces the FN rate of SLNB itself and is currently the most popular procedure for determining the stage of the axilla in this group of patients. Our analysis of all patients who underwent SLN and TAD shows that evaluation of the clipped node reduced the FN rate by 0.0% compared with 6.9% for SLN alone. This technique has recently been introduced in the main guidelines and is associated with a further reduction in FNR, ranging from 2% to 6.8%.^8^, ^9^, ^10^

In essence, breast cancers and breast pathologies in general remain vital and controversial issues today,^11^, ^12^, ^13^, ^14^, ^15^ and we found that we successfully improved the accuracy of axillary staging in clinically positive patients with lymph nodes who received neoadjuvant therapy by performing TAD, particularly when SLNB was negative. Still, the marked MS node was positive for metastasis.

### Limitation

This study was limited to a single comprehensive oncology facility with specialized breast radiologists who had considerable expertise in ultrasonography, resulting in accurate diagnoses and the subsequent introduction of magnetic beads into the biopsied lymph node. In addition, it possesses a small sample size, a single-center design, a lack of an ALND gold standard, and an absence of long-term survival/morbidity data.

## Conclusions

Neoadjuvant Therapy (NACT) is paramount, as it enables more adequate surgical procedures targeted at individual patients, with the expectation of improving the prognosis of oncological diseases. This applies both to the breast, where partial surgery can be performed and an initially inoperable finding can be made operable, and to the reduction of the extent of surgery in the axilla. NACT therapy also provides sufficient time for genetic testing and allows for the planning of reconstructive surgery. The combination of SLNB and the removal of this clipped node (TAD) is crucial for avoiding complete axillary dissection in patients with ycN0 after NACT, while simultaneously reducing the risk of false negativity of the SLN, which leads to a reduction in morbidity in oncological patients.

## Authors’ contributions

Daniel Toman: Conceptualization; Data curation; Formal analysis; Investigation; Methodology; Project administration; Resources; Software; Validation; Visualization; Writing-original draft.

Ilker Sengul: Formal analysis; Investigation; Software; Supervision; Validation; Visualization; Writing-review & editing.

Anton Pelikán: Formal analysis; Investigation; Methodology; Project administration; Software; Validation; Visualization.

Demet Sengul: Formal analysis; Investigation; Software; Supervision; Validation; Visualization; Writing-review & editing.

Otakar Kubala: Formal analysis; Investigation; Methodology; Project administration; Validation; Visualization.

Jiří Prokop: Investigation; Methodology; Project administration; Validation; Visualization.

Markéta Pernicová: Investigation; Project administration; Validation; Visualization.

Lubomír Tulinský: Investigation; Methodology; Project administration; Validation; Visualization.

Peter Ihnát: Investigation; Methodology; Project administration; Validation; Visualization.

José Maria Soares-Junior: Investigation; Software; Supervision; Validation; Writing-review & editing.

## Declaration of competing interest

The authors declare no conflicts of interest.

## Data Availability

The datasets used and/or analyzed during this study are available from the corresponding author upon reasonable request.
